# P-1349. Multi-hospital Dissemination of Ampicillin Resistance Associated with a Frame-shift Deletion in *pbp4* of Vancomycin-Resistant *Enterococcus faecalis* (VREfs)

**DOI:** 10.1093/ofid/ofae631.1526

**Published:** 2025-01-29

**Authors:** Lorena Diaz, Kavindra Singh, Truc Cecilia Tran, Valeria E Quiroz, Katherine Soto, Anne S Peters, Rodrigo de Paula Baptista, Jose R W Martínez, Juan Ugalde, Guillermo Hoppe, Araos Rafael, Maria Paz Acuna, Ignacio Martinez, Patricia Garcia, William R Miller, José M Munita, Cesar A Arias, Diana Panesso

**Affiliations:** Univerdidad del Desarrollo, Santiago, Region Metropolitana, Chile; Houston methodist research institute, Houston, Texas; Houston Methodist Research Institute, Houston, TX; Universidad del Desarrollo, Santiago, Region Metropolitana, Chile; Clínica Alemana - Universidad del Desarrollo - Millennium Initiative for Collaborative Research On Bacterial Resistance (MICROB-R), Santiago, Region Metropolitana, Chile; Universidad del Desarrollo, Santiago, Region Metropolitana, Chile; Houston Methodist Hospital, Houston, Texas; Genomics & Resistant Microbes (GeRM), Instituto de Ciencias e Innovación en Medicina, Facultad de Medicina Clínica Alemana, Universidad del Desarrollo, Chile; Millennium Initiative for Collaborative Research on Bacterial Resistance (MICROB-R), Santiago, Region Metropolitana, Chile; Universidad Andrés Bello - Millennium Initiative for Collaborative Research On Bacterial Resistance (MICROB-R), Santiago, Region Metropolitana, Chile; Houston Methodist Research Institute / Weill Cornell Medical College, Houston, Texas; Universidad del Desarrollo, Santiago, Region Metropolitana, Chile; Hospital La Florida, Santiago, Region Metropolitana, Chile; Hospital La Florida, Santiago, Region Metropolitana, Chile; Pontificia Universidad Catolica de Chile, Santiago, Region Metropolitana, Chile; Houston Methodist Research Institute, Houston, TX; Clínica Alemana - Universidad del Desarrollo, Santiago, Chile; Houston Methodist and Weill Cornell Medical College, Houston, TX; Houston Methodist Research Institute, Houston, TX

## Abstract

**Background:**

Penicillin (PEN) and ampicillin (AMP) resistance is rare in *E. faecalis,* and may arise via increased expression of penicillin-binding protein 4 (PBP4), mutations affecting PBP4’s affinity to β-lactams, and rarely β-lactamase production. We recently identified 23 *vanA* vancomycin-resistant *E. faecalis* (VREfs), recovered from 19 patients in 4 hospitals in Chile (2020-2023) that exhibited PEN resistance and unusually elevated AMP MICs (4-16 µg/mL). Here, we characterize the molecular basis of the reduced β-lactam susceptibility in these VREfs isolates.

**Table 1.**
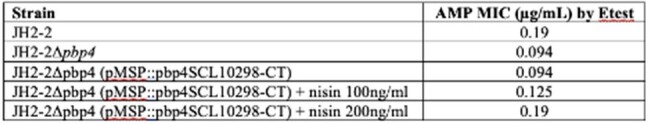
AMP susceptibility after inducible expression of pbp4SCL10298 in the pbp4-defective and AMP hypersusceptible strain JH2-2Δpbp4

**Methods:**

We analyzed 23 VREfs including sequence type (ST), resistome, PBP sequences and *pbp4* promoter. PBP4 production was assessed by ELISA and Western blot using goat anti-rPBP4 sera, in the Chilean VREfs isolate SCL10298 and using *pbp4*-defective strains (JH2-2Δ*pbp4* and OG117Δ*pbp4*). We recreated the *pbp4* changes of SCL10298 by allelic replacement of *E. faecalis* OG117 (OG117

Δ*pbp4*_SCL10298_) using CRISPR-Cas9. Expression of *pbp4*_SCL10298_ was also assessed in-trans in a JH2-2 background (JH2-2Δ*pbp4*) using a nisin-inducing vector (pMSP3535).

**Figure 1.**
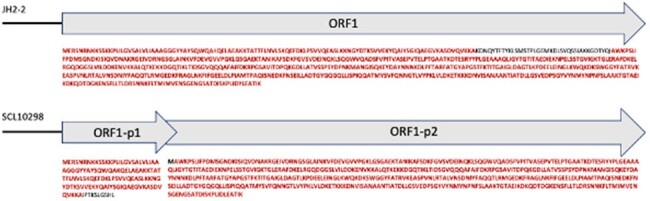
Schematic representation of predicted open reading frames (ORFs) on the JH2-2 and SCL10298

Black line represents the nucleotide sequence for the PBP4 promoter region, followed by the predicted ORFs indicated with grey arrows. Text in red represents the amino acid sequence sharing 100% identity between JH2-2 ORF1 and SCL10298 ORF1-p1 and ORF1-p2.

**Results:**

All 23 isolates belonged to ST1518 (an ST6 single-locus variant). No β-lactamase-encoding genes or changes in *pbp*promoter regions were detected. A frameshift deletion of 100 nt was observed in *pbp4* in all isolates, resulting in 2 open reading frames, including a potential transcript of a shorter *pbp4* (ORF1-p1, fig1). All the other PBP sequences were identical to those of *E. faecalis* JH2-2. PBP4 was detected in SCL10298 by whole-cell ELISA. Further, western blots revealed no differences in PBP4 amounts between SCL10298 and control strains. Allelic replacement of OG117Δ did not result in decreased AMP susceptibility as compared to the parental OG117 strain (AMP MIC 0.5 µg/mL) nor did the inducible expression of the truncated version of *pbp4* in JH2-2Δ*pbp4* (0.19 µg/ml, table1).

**Conclusion:**

We document the emergence of a new lineage of VREfs in Chile with alteration in PBP4 that exhibits decreased susceptibility to B-lactams (akin *E. faecium*). Our molecular experiments suggest that an alteration of PBP4 is necessary, but not sufficient to increase the MICs of AMP. Therefore, we propose the strain background might be key for the full expression of the B-lactam-resistant phenotype.

**Disclosures:**

**William R. Miller, M.D.**, Merck: Grant/Research Support|UptoDate: Royalties **José M Munita, MD**, MSD: Grant/Research Support|Pfizer: Grant/Research Support

